# SUMOylation of the ubiquitin ligase IDOL decreases LDL receptor levels and is reversed by SENP1

**DOI:** 10.1074/jbc.RA120.015420

**Published:** 2020-11-23

**Authors:** Ju-Qiong Wang, Zi-Cun Lin, Liang-Liang Li, Shao-Fang Zhang, Wei-Hui Li, Wei Liu, Bao-Liang Song, Jie Luo

**Affiliations:** Hubei Key Laboratory of Cell Homeostasis, College of Life Sciences, Wuhan University, Wuhan, China

**Keywords:** low-density lipoprotein (LDL), receptor endocytosis, ubiquitin ligase, posttranslational modification (PTM), SUMOylation, LDL receptor, inducible degrader of the low-density lipoprotein receptor (IDOL), ubiquitination, SUMO-specific peptidase, deSUMOylation, AFC, 7-amino-4-trifluoromethylcoumarin, AMC, 7-amino-4-methylcoumarin, DiI, 1,1'-dioctadecyl-3,3,3',3'-tetramethylindocarbocyanine perchlorate, IDOL, inducible degrader of the low-density lipoprotein receptor, LDL, low-density lipoprotein, LDL-C, low-density lipoprotein-cholesterol, LDLR, low-density lipoprotein receptor, LXR, liver X receptor, PCSK9, proprotein convertase subtilisin/kexin type 9, RING, really interesting new gene, SENP, SUMO-specific peptidase, SREBP, sterol regulatory element-binding protein, SUMO, small ubiquitin-like modifier, USP, ubiquitin-specific protease, WT, wild-type

## Abstract

Inducible degrader of the low-density lipoprotein receptor (IDOL) is an E3 ubiquitin ligase mediating degradation of low-density lipoprotein (LDL) receptor (LDLR). IDOL also controls its own stability through autoubiquitination, primarily at lysine 293. Whether IDOL may undergo other forms of posttranslational modification is unknown. In this study, we show that IDOL can be modified by small ubiquitin-like modifier 1 at the K293 residue at least. The SUMOylation of IDOL counteracts its ubiquitination and augments IDOL protein levels. SUMOylation and the associated increase of IDOL protein are effectively reversed by SUMO-specific peptidase 1 (SENP1) in an activity-dependent manner. We further demonstrate that SENP1 affects LDLR protein levels by modulating IDOL. Overexpression of SENP1 increases LDLR protein levels and enhances LDL uptake in cultured cells. On the contrary, loss of SENP1 lowers LDLR levels in an IDOL-dependent manner and reduces LDL endocytosis. Collectively, our results reveal SUMOylation as a new regulatory posttranslational modification of IDOL and suggest that SENP1 positively regulates the LDLR pathway *via* deSUMOylation of IDOL and may therefore be exploited for the treatment of cardiovascular disease.

The low-density lipoprotein (LDL) receptor (LDLR)-mediated uptake of circulating LDL particles is a prototype of receptor-mediated endocytosis and plays a key role in regulating cholesterol homeostasis at both cellular and whole-body levels (1). The LDLR pathway begins with LDL binding to LDLR, followed by clathrin-dependent internalization of the LDL–LDLR complex. In the acidic endosomes, LDLR is induced to dissociate from LDL, allowing the latter to be further delivered to late endosomes/lysosomes, where LDL-carried cholesteryl esters are hydrolyzed to release cholesterol (2). LDLR is either directed to the cell surface for reutilization or targeted to lysosome for degradation. Mutations in *LDLR* as well as genes encoding apolipoprotein B and LDLR adaptor protein 1 (also known as autosomal recessive hypercholesterolemia), which are involved in LDL–LDLR binding and LDL–LDLR complex endocytosis, respectively, confer elevated levels of plasma LDL-cholesterol (LDL-C) that eventually increase the risk for cardiovascular disease (3, 4). The gain-of-function mutations in proprotein convertase subtilisin/kexin type 9 (*PCSK9*)—which binds and targets LDLR for lysosomal degradation—also underlie a subclass of familial hypercholesterolemia. Blocking PCSK9-mediated LDLR degradation using the anti-PCSK9 monoclonal antibodies has emerged as an effective strategy to lower LDL-C levels in familial hypercholesterolemia patients or those intolerant to statins (5).

Inducible degrader of the LDLR (IDOL, also known as myosin regulatory light-chain interacting protein) is another critical regulator of the LDLR pathway. As an E3 ubiquitin ligase, IDOL binds the cytoplasmic tail of LDLR *via* the N-terminal FERM 3b subdomain and triggers K48- and K63-linked polyubiquitination *via* the C-terminal really interesting new gene (RING) domain (6, 7, 8). The ubiquitinated LDLR is internalized in an epsin-dependent manner, sorted to multivesicular bodies by the endosomal sorting complexes required for transport complexes, and finally degraded in lysosomes (9, 10). Aside from LDLR, IDOL can modulate its own stability through formation of a homodimer followed by autoubiquitination and degradation in proteasomes (6, 8, 11). Disruption of IDOL dimerization between the RING domain and deubiquitination of IDOL by ubiquitin-specific protease (USP) 2 prevent IDOL degradation as well as abolish its ability to degrade LDLR (11, 12). Another dimerization-defective IDOL G51S mutation that has been associated with high blood LDL-C levels in humans can increase IDOL protein abundance and, notably, accelerate IDOL-induced LDLR degradation (13). These results highlight the importance of IDOL stabilization in modulating LDLR expression and blood LDL-C levels.

SUMOylation resembles ubiquitination in that substrate proteins are covalently modified with small molecules in single entities or polymeric chains *via* the E1-E2-E3 enzymatic cascade (14, 15). However, unlike simple addition of ubiquitin to target proteins, small ubiquitin-like modifier (SUMO, also called sentrin)—which comprises five paralogues in mammals—is initially synthesized as a precursor and undergoes proteolytic processing to become active (16, 17). The maturation of SUMO1–3 requires a C-terminal cleavage mediated by SUMO-specific peptidases (SENPs) (18). Humans have six SENPs that exhibit distinct substrate preferences and subcellular distributions (19, 20). These cysteine proteases can also reverse SUMOylation by deconjugating SUMO from substrates. The SUMOylation–deSUMOylation cycle governs many biological processes by affecting protein stability, activity, localization as well as their interaction with other proteins (21). Deregulation of SUMOylation or deSUMOylation has been implicated in various cancers and neurodegenerative diseases (22, 23). However, there has been a paucity of reports on how SUMO modification modulates cholesterol metabolism. The nuclear form of sterol regulatory element-binding protein (SREBP) 2, the master transcriptional regulator of cholesterol biosynthesis and uptake, can be SUMOylated at the K464 residue for decreased transcriptional activity (24). SUMOylation is also reported to promote the nuclear receptor liver receptor homolog 1 to interact with its co-repressor prosperso homeobox protein 1, leading to reduced transcription of the target genes involved in reverse cholesterol transport (25). Whether SUMO can modify and regulate other players of the cholesterol pathway is largely unknown.

In the current study, we report for the first time that IDOL is a SUMO1 target protein. SUMOylation occurs at multiple lysine residues including K293, which is also a key ubiquitination site. SUMOylation stabilizes IDOL by competing against its autoubiquitination, thus increasing IDOL protein level and its potency in degrading LDLR. Moreover, we show that SENP1 can deSUMOylate and destabilize IDOL. Overexpression of SENP1 increases LDLR protein level and LDL uptake, whereas knockdown or knockout of SENP1 has opposite effects. Together, these results reveal SUMOylation as a new posttranslational modification that modulates IDOL abundance and suggest a role of SENP1 in regulating the LDLR pathway.

## Result

### IDOL is modified by SUMO1 primarily at the K293 residue

**Figure 1:**
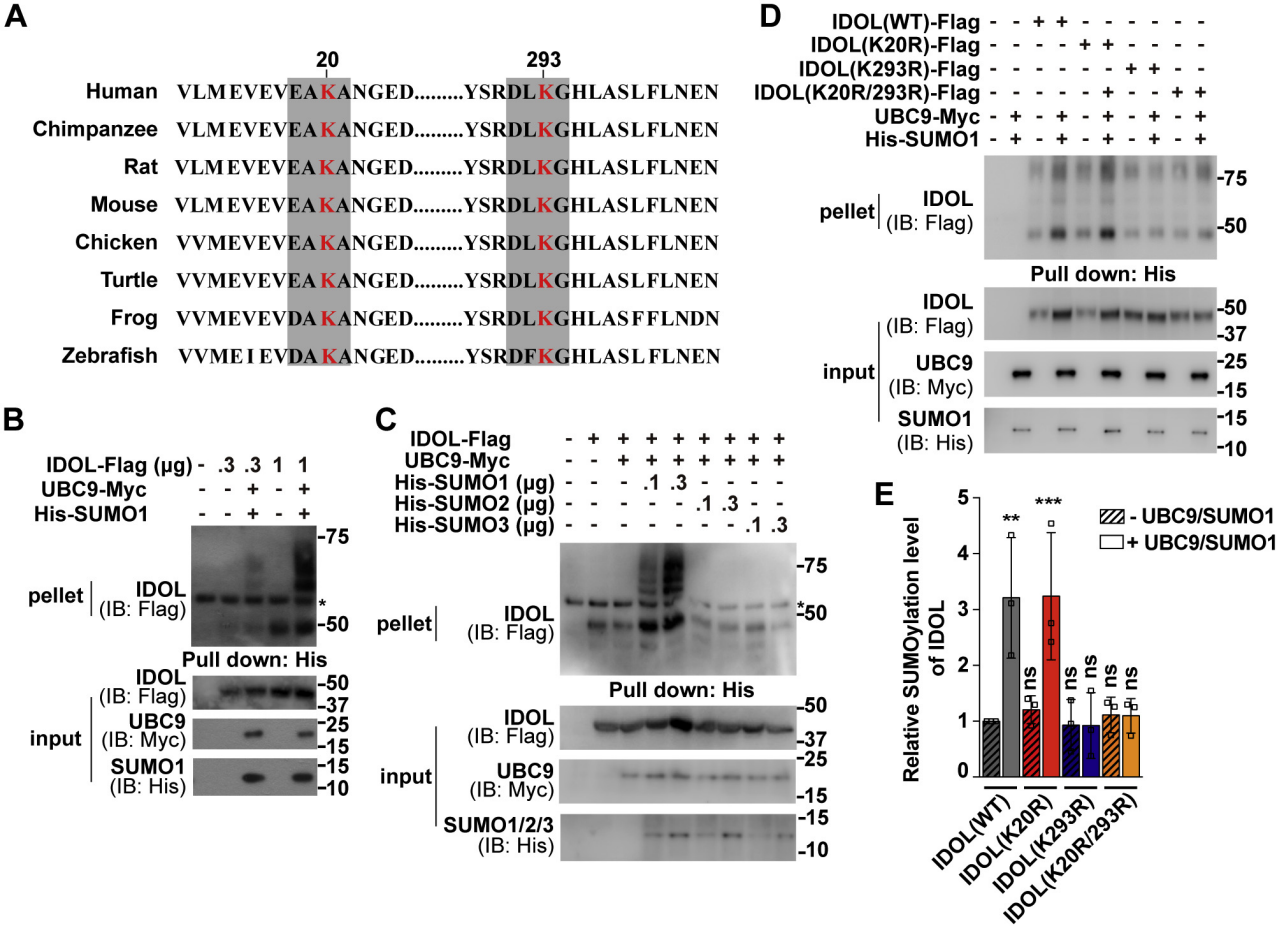
**IDOL is conjugated with SUMO1 primarily at the K293 residue.***A*, alignment of the IDOL sequence across species with two inverted SUMOylation consensus motifs E/DxKψ (where x stands for any amino acid and ψ for a hydrophobic amino acid) shaded in gray. The K20 and K293 residues are in red. *B*–*D*, Huh7 cells were transfected with the indicated plasmids. After 48 h, cells were treated with 10 μM MG132 for 3 h and harvested. Lysates were pulled down by Ni-NTA agarose and then subjected to immunoblotting with the indicated antibodies. Asterisks indicate nonspecific bands. *E*, densitometric analysis of the high-molecular-weight smears relative to the unmodified IDOL band as shown in (*D*). Values are normalized to the value obtained from cells transfected with the wild-type form of IDOL only and presented as mean ± SEM (n = 3 independent experiments). ∗∗*p* < 0.01, ∗∗∗*p* < 0.001, ns, no significance. One-way ANOVA. Results in (*B*) and (*C*) are representative of two independent experiments and in (*D*) are of three independent experiments. IB, immunoblotting; IDOL, inducible degrader of the low-density lipoprotein receptor; SUMO, small ubiquitin-like modifier; WT, wild-type.

SUMOylation can occur on a lysine residue within the inverted SUMOylation consensus motif E/DxKψ (where x stands for any amino acid and ψ for a hydrophobic amino acid) (26). Analysis of the human IDOL sequence revealed EAK_20_A and DLK_293_G, both of which are highly conserved in vertebrates, that matched the criteria (Fig. 1*A*). To examine whether IDOL is modified by SUMO, we transiently transfected human hepatocellular carcinoma Huh7 cells with the plasmid expressing Flag-tagged IDOL alone or together with the plasmids expressing Myc-tagged UBC9, the SUMO-conjugating enzyme E2, and His-tagged SUMO1. Lysates were pulled down by the nickel beads and examined for the presence of IDOL. We detected a dose-dependent increase in the high-molecular-weight smears when SUMOylation machinery was present (Fig. 1*B*). However, substitution of SUMO2 or SUMO3 for SUMO1 yielded negative results (Fig. 1*C*). These data suggest that IDOL is specifically conjugated with SUMO1.

To determine the potential SUMOylation site(s) on IDOL, we performed the SUMOylation assay using IDOL mutants in which K20 and K293 were individually or simultaneously replaced by arginine. Contrasting to the wild-type (WT) form of IDOL that was extensively modified by SUMO1, the K293R and K20R/K293R mutants were apparently less SUMOylated, whereas the K20R mutant had a similar level of SUMO1 conjugates (Fig. 1*D*). Quantitative analysis showed that the K293R mutation indeed caused significant decreases in IDOL SUMOylation (Fig. 1*E*). These results suggest that IDOL is SUMOylated by SUMO1 mainly at the K293 residue. However, other SUMOylation site(s) might also exist because the K293R mutation did not completely abolish SUMO1-conjugation of IDOL.

### SUMOylation elevates IDOL protein levels by inhibiting its autoubiquitination

**Figure 2:**
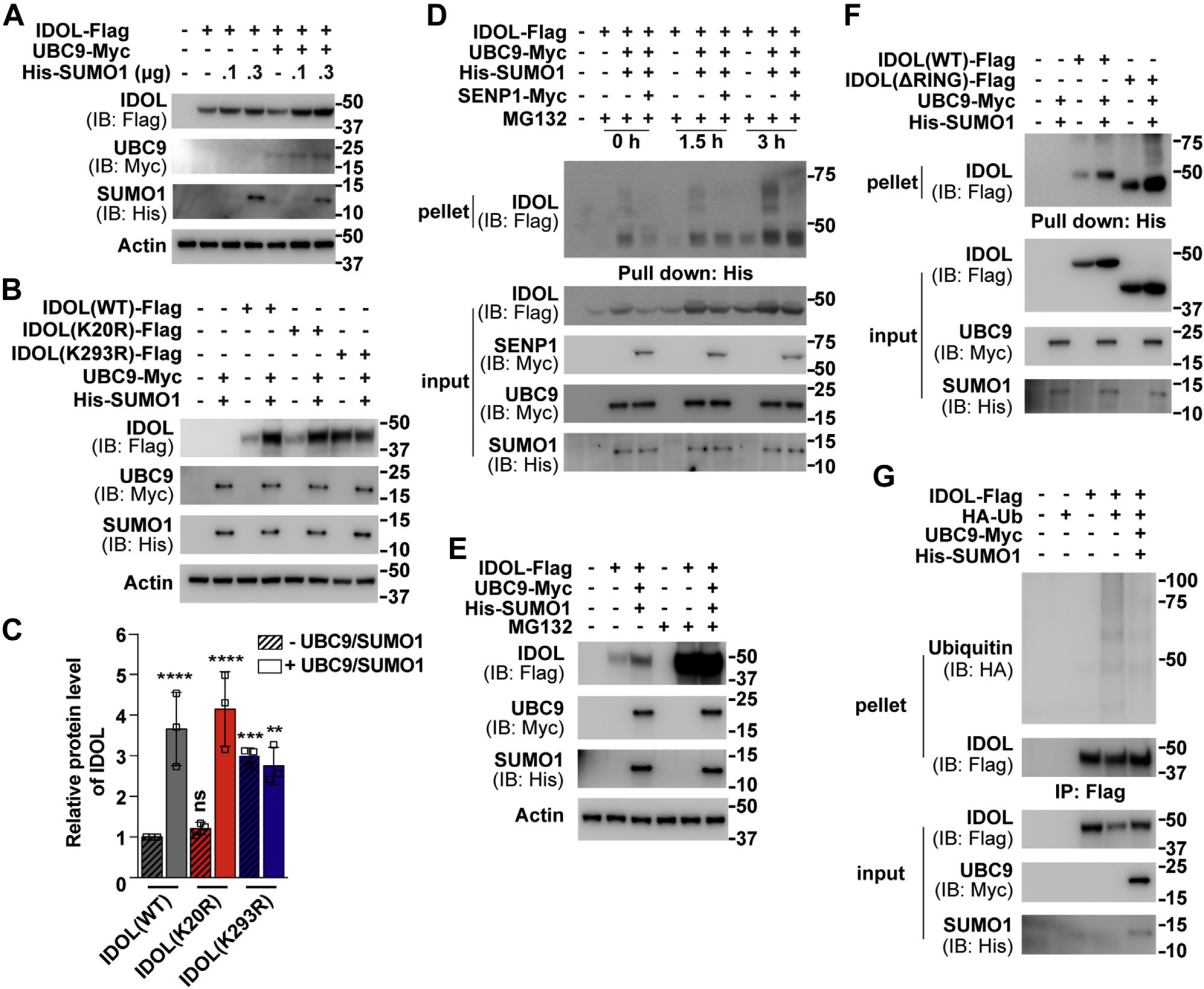
**SUMOylation and ubiquitination competitively regulate IDOL level.***A*–*B*, Huh7 cells were transfected as indicated. After 48 h, cells were harvested for immunoblotting analysis. *C*, densitometric analysis of the IDOL bands in (*B*). Values are normalized to the value obtained from cells transfected with the wild-type form of IDOL only and presented as mean ± SEM (n = 3 independent experiments). ∗∗*p* < 0.01, ∗∗∗*p* < 0.001, ∗∗∗∗*p* < 0.0001, ns, no significance. One-way ANOVA. *D*, Huh7 cells were transfected as indicated. After 48 h, cells were treated with 10 μM MG132 for the indicated periods and harvested for the SUMOylation assay as described in Figure 1*E*, Huh7 cells were transfected with as indicated. After 48 h, cells were treated with or without 10 μM MG132 for 3 h and harvested for immunoblotting analysis. *F*, Huh7 cells were transfected as indicated. After 48 h, cells were treated with 10 μM MG132 for 3 h and harvested for the SUMOylation assay as described in Figure 1*G*, Huh7 cells were transfected as indicated. After 48 h, cells were treated with 10 μM MG132 for 3 h and harvested. Lysates were immunoprecipitated (IPed) with anti-FLAG M2 agarose beads and then subjected to IB with the indicated antibodies. Results in (*A*), (*B*), (*E*), and (*G*) are representative of three independent experiments. Results in (*D*) and (*F*) are representative of two independent experiments. IB, immunoblotting; IDOL, inducible degrader of the low-density lipoprotein receptor; SENP, SUMO-specific peptidase; SUMO, small ubiquitin-like modifier; WT, wild-type.

IDOL is unstable, and the overexpressed IDOL protein has a half-life of about 2 h (8, 13). We next sought to investigate whether IDOL SUMOylation may affect its protein abundance. Because there have been no commercial antibodies that can effectively detect endogenous IDOL (10, 12), we examined the level of transfected protein in the absence or presence of UBC9 and SUMO1 in Huh7 cells. The expression of IDOL protein was proportionally elevated with increasing concentrations of SUMO1 (Fig. 2*A*). Single mutation of the K20 residue failed to alter the responsiveness of IDOL to UBC9 and SUMO1, whereas arginine substitution of the K293 residue, which is also the primary ubiquitination site (6), resulted in an increased basal level of IDOL protein that was resistant to further augmentation by UBC9 and SUMO1 (Fig. 2, *B*–*C*).

We next treated Huh7 cells transfected with IDOL and UBC9 plus SUMO1 with the proteasome inhibitor MG132 to exclude the possibility that enhanced IDOL protein expression may result from reduced ubiquitination. MG132 caused parallel increases in the amounts of SUMOylated (Fig. 2*D*) and total (Fig. 2*E*) IDOL proteins. SENP1 that can deconjugate SUMO1-modified proteins (20, 27) effectively eliminated the bands higher than the predicted size of unmodified IDOL protein (Fig. 2*D*). Deletion of the entire RING domain, which contributes to IDOL autoubiquitination and degradation *via* several mechanisms (6, 8, 11), elevated IDOL SUMOylation as well (Fig. 2*F*). These results imply a possible competition between the two posttranslational modifications.

To directly demonstrate that IDOL SUMOylation can antagonize its ubiquitination, we co-expressed IDOL and ubiquitin in the absence or presence of UBC9 plus SUMO1 in Huh7 cells. As shown in Figure 2*G*, ubiquitination of IDOL was greatly reduced when SUMOylation components were provided. These results suggest that SUMOylation and ubiquitination regulate IDOL protein levels in a competitive manner.

### SENP1 deSUMOylates IDOL and decreases its protein level

**Figure 3:**
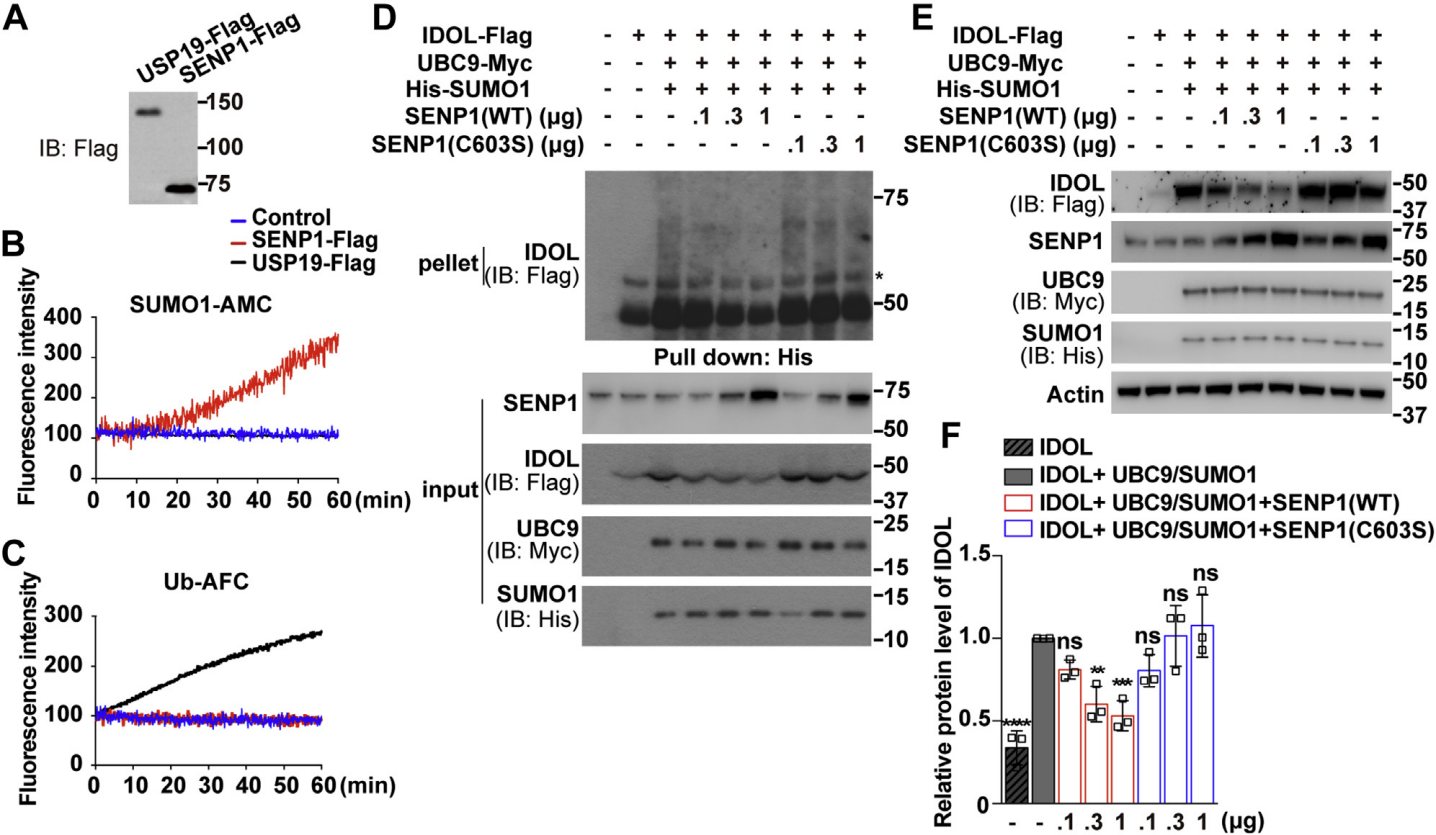
**SENP1 deSUMOylates IDOL and reduces IDOL abundance.***A*, HEK293T cells were transfected with plasmids expressing Flag-tagged SENP1 or USP19. After 48 h, cells were harvested and IPed with anti-FLAG M2 agarose beads. Bound proteins were eluted by 3×FLAG peptides and analyzed by immunoblotting. *B*–*C*, equal amounts of SENP1 or USP19 in TBS (vehicle control) were incubated with 1 μM SUMO1-AMC (*B*) or 0.5 μM ubiquitin (Ub)-AFC (*C*), and released fluorescence of free AMC or AFC was analyzed. *D*, Huh7 cells were transfected as indicated. After 48 h, cells were treated with 10 μM MG132 for 3 h and harvested for the SUMOylation assay as described in Figure 1. Asterisk indicates nonspecific bands. *E*, Huh7 cells were transfected as indicated. After 48 h, cells were harvested for immunoblotting analysis. *F*, densitometric analysis of the IDOL bands in (*E*). Values are normalized to the value obtained from cells transfected with IDOL and UBC9 plus SUMO1 and presented as mean ± SEM (n = 3 independent experiments). ∗∗*p* < 0.01, ∗∗∗*p* < 0.001, ∗∗∗∗*p* < 0.0001, ns, no significance. One-way ANOVA. Results in (*A*), (*B*), and (*C*) are from a single experiment. Results in (*D*) are representative of two independent experiments and in (*E*) are of three independent experiments. AFC, 7-amino-4-trifluoromethylcoumarin; AMC, 7-amino-4-methylcoumarin; IB, immunoblotting; IDOL, inducible degrader of the low-density lipoprotein receptor; SENP, SUMO-specific peptidase; SUMO, small ubiquitin-like modifier; USP, ubiquitin-specific protease; WT, wild-type.

To further investigate the effects of SENP1 on IDOL, we prepared the recombinant SENP1-Flag protein from HEK293T cells (Fig. 3*A*). The deubiquitinating enzyme USP19 that deconjugates ubiquitin but not SUMO1 was included as a control. The Flag-tagged SENP1 displayed a high potency in hydrolyzing SUMO1-AMC (7-amino-4-methylcoumarin) as the substrate (Fig. 3*B*). As expected, USP19 but not SENP1 was able to hydrolyze ubiquitin-AFC (7-amino-4-trifluoromethylcoumarin) (Fig. 3*C*). We next applied increasing concentrations of WT or catalytically inactive (C603S) SENP1 in the IDOL SUMOylation assay. The SUMO1-modified species of IDOL were dose-dependently reduced by the active SENP1 but not the C603S mutant (Fig. 3*D*). The level of IDOL protein was significantly reduced by higher concentrations of WT SENP1 but remained unresponsive to the inactive SENP1 (Fig. 3, *E*–*F*). So far, our results suggest that SUMO1-mediated SUMOylation and SENP1-mediated deSUMOylation are two opposing processes regulating IDOL protein levels.

### SENP1-mediated deSUMOylation of IDOL increases LDLR expression and LDL uptake

**Figure 4:**
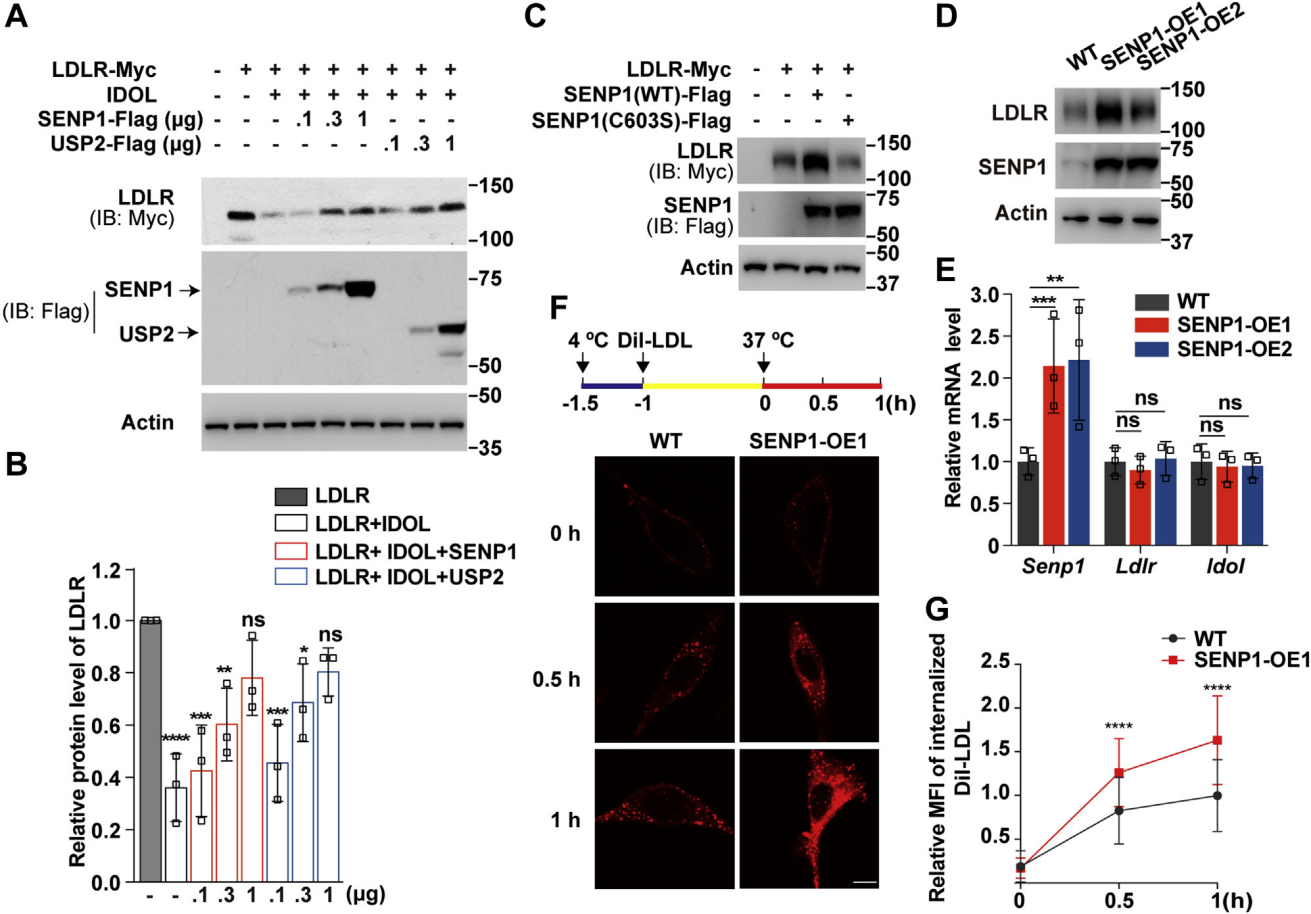
**SENP1 counteracts IDOL-induced degradation of LDLR and augments LDLR-dependent endocytosis of LDL.***A*, Huh7 cells were transfected as indicated. After 32 h, cells were subjected to cholesterol depletion by incubating with medium A plus 5% lipoprotein-deficient serum, 1 μM lovastatin, and 10 μM mevalonate for 16 h. Cells were then harvested for immunoblotting analysis. *B*, densitometric analysis of the LDLR bands in (*A*). Values are normalized to the value obtained from cells transfected with LDLR only and presented as mean ± SEM (n = 3 independent experiments). ∗*p* < 0.05, ∗∗*p* < 0.01, ∗∗∗*p* < 0.001, ∗∗∗∗*p* < 0.0001, ns, no significance. One-way ANOVA. *C*, Huh7 cells were transfected as indicated. After 32 h, cells were subjected to cholesterol depletion for 16 h and then harvested for immunoblotting analysis. *D*–*E*, CRL1601 cells (WT) and two clones of CRL1601 cells stably expressing SENP1-Flag (SENP1-OE1 and SENP1-OE2) were subjected to cholesterol depletion for 16 h and then harvested for immunoblotting (*D*) or RT-qPCR analysis (*E*). Data are normalized to WT cells and presented as mean ± SEM (n = 3 independent experiments). ∗∗*p* < 0.01, ∗∗∗*p* < 0.001, ns, no significance. One-way ANOVA. *F*, CRL1601 and SENP1-OE1 cells were depleted of cholesterol at 4 °C for 30 min and then incubated with 10 μg/ml Dil-low-density lipoprotein (LDL) at 4 °C for 1 h. Cells were rinsed with PBS and cultured in cholesterol depletion medium at 37 °C for the indicated time points. Scale bar, 20 μm. *G*, Quantification of the relative MFI of internalized DiI-LDL in (*F*). The relative MFI of internalized DiI-LDL in WT cells at 1 h is defined as 1. Data are presented as mean ± SEM (n = 100 cells from 2 independent experiments). ∗∗∗∗*p* < 0.0001. Unpaired two-tailed Student’s *t* test. Results in (*A*) and (*C*) are representative of three independent experiments. Results in (*D*) and (*F*) are representative of two independent experiments. IB, immunoblotting; IDOL, inducible degrader of the low-density lipoprotein receptor; LDLR, low-density lipoprotein receptor; MFI, mean fluorescence intensity; ns, no significance; SENP, SUMO-specific peptidase; WT, wild-type.

IDOL negatively modulates LDLR stability by targeting its degradation in lysosomes (6, 7, 8). We next sought to determine whether SENP1 affects IDOL-mediated degradation of LDLR. USP2 can counteract the degradative effect of IDOL on LDLR (12) and was used as a control. As shown in Figure 4, *A*–*B*, SENP1 prevented degradation of the transfected LDLR protein induced by IDOL to a similar extent as USP2. Inactivation of the protease activity completely abolished the stimulatory effect of SENP1 on LDLR (Fig. 4*C*). We also generated two individual lines of CRL1601 cells stably expressing SENP1 (hereinafter refer to as the stable cells). We found these cells had elevated levels of endogenous LDLR protein compared with the parental cells (Fig. 4*D*). The mRNA abundance of *Ldlr* and *Idol* was not altered by SENP1 overexpression (Fig. 4*E*).

PCSK9 is another potent LDLR degrader aside from IDOL (28, 29). To rule out the possibility that PCSK9 was involved in LDLR elevation upon SENP1 overexpression, CRL1601 and stable cells were incubated with the purified PCSK9 protein at various concentrations in cholesterol-depleting medium containing lipoprotein-deficient serum, lovastatin and minimal amount of mevalonate, a condition in which the SREBP pathway and thus LDLR expression are activated (2). Cells grown under normal cholesterol-rich conditions were used as controls. Despite higher basal levels of LDLR in the stable cells, PCSK9 was capable of inducing LDLR degradation at a similar rate as in WT cells (Fig. S1).

We next examined the effects of SENP1 on LDLR-mediated uptake of DiI (1,1′-dioctadecyl-3,3,3′,3′-tetramethylindocarbocyanine perchlorate)-labeled LDL. CRL1601 and stable cells were deprived of cholesterol and pulsed with DiI-LDL for 1 h. Cells were then shifted to 37 °C to allow DiI-LDL endocytosis to occur. We observed time-dependent increases of DiI-positive puncta in the cytosol of both control and stable cells (Fig. 4*F*). However, the internalization of DiI-LDL was much faster in the stable cells than that in control cells (Fig. 4*G*).

**Figure 5:**
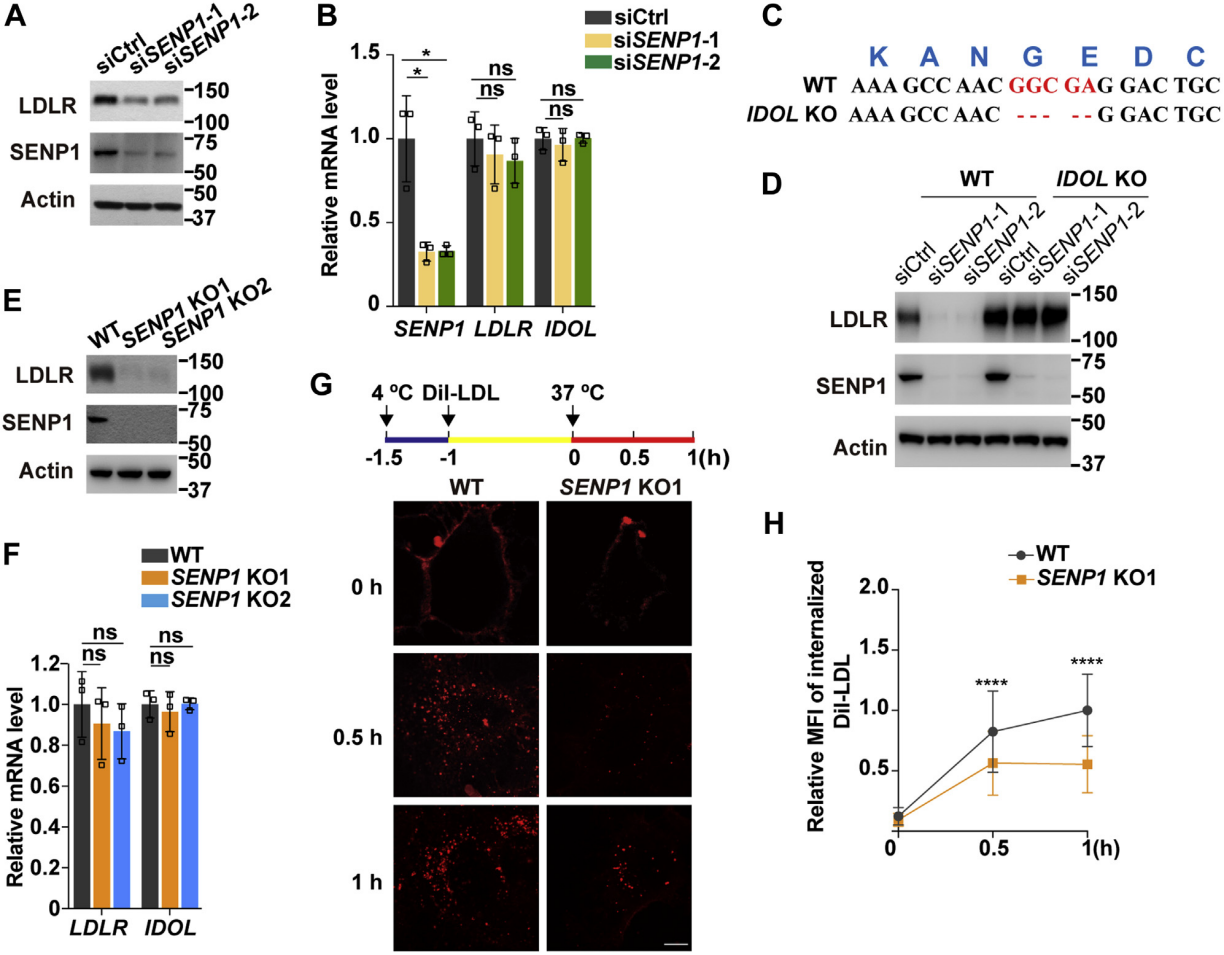
**SENP1 deficiency reduces LDLR expression and LDL endocytosis.***A*, Huh7 cells were transfected with scrambled siRNAs (siCtrl) or two individual siRNAs targeting *SENP1* (si*SENP1*-1 and si*SENP1*-2). After 32 h, cells were depleted of cholesterol for 16 h and harvested for immunoblotting (*A*) or RT-qPCR analysis (*B*). Data are normalized to control cells and presented as mean ± SEM (n = 3 independent experiments). ∗*p* < 0.05, ns, no significance. One-way ANOVA. *C*, Sanger sequencing analysis of *IDOL* knockout (KO) cells bearing a frameshift mutation in the exon 1 of the *IDOL* gene. *D*, Huh7 and *IDOL* KO cells were transfected with scrambled siRNAs or two individual siRNAs targeting *SENP1*. After 32 h, cells were depleted of cholesterol for 16 h and harvested for immunoblotting. *E*–*F*, two lines of Huh7 cells lacking *SENP1* (*SENP1* KO1 and *SENP1* KO2) were depleted of cholesterol for 16 h and harvested for immunoblotting (*E*) or RT-qPCR analysis (*F*). Data are normalized to control cells and presented as mean ± SEM (n = 3 independent experiments). ns, no significance. One-way ANOVA. *G*, Huh7 cells and *SENP1* KO1 cells were treated as depicted in Figure 4*F*. Scale bar, 20 μm. *H*, Quantification of the relative MFI of internalized DiI-LDL in (*G*). The relative MFI of internalized DiI-LDL in WT cells at 1 h is defined as 1. Data are presented as mean ± SEM (n = 100 cells from 2 independent experiments). ∗∗∗∗*p* < 0.0001. Unpaired two-tailed Student’s *t* test. Results in (*A*) and (*D*) are representative of three independent experiments. Results in (*E*) and (*G*) are representative of two independent experiments. IDOL, inducible degrader of the low-density lipoprotein receptor; LDL, low-density lipoprotein; LDLR, low-density lipoprotein receptor; MFI, mean fluorescence intensity; SENP, SUMO-specific peptidase; WT, wild-type.

To corroborate the positive role of SENP1 on LDLR levels and LDL uptake, we knocked down *SENP1* in Huh7 cells with two different small interfering RNA duplexes (si*SENP1*-1, si*SENP1*-2). Contrary to the findings under the conditions where SENP1 was overexpressed (Fig. 4), silencing of *SENP1* decreased the endogenous LDLR protein level (Fig. 5*A*) without affecting the mRNA expression of *LDLR* or *IDOL* (Fig. 5*B*). Of note, *IDOL* knockout cells (Fig. 5*C*) had constitutively high levels of LDLR protein even when *SENP1* was depleted (Fig. 5*D*). These results suggest an absolute requirement of IDOL for LDLR downregulation upon SENP1 deficiency. Consistently, in CRISPR/Cas9-mediated *SENP1* knockout cells, we detected profoundly reduced expression of LDLR protein but not that of *LDLR* or *IDOL* mRNA (Fig. 5, *E*–*F*). The pulse-chase experiment showed that in *SENP1* knockout cells, the internalized DiI-LDL was about half of that in control cells following a 1-h pulse (Fig. 5, *G–H*). Altogether, these results demonstrate that SENP1 increases LDLR protein and LDL uptake through deSUMOylating IDOL.

## Discussion

**Figure 6:**
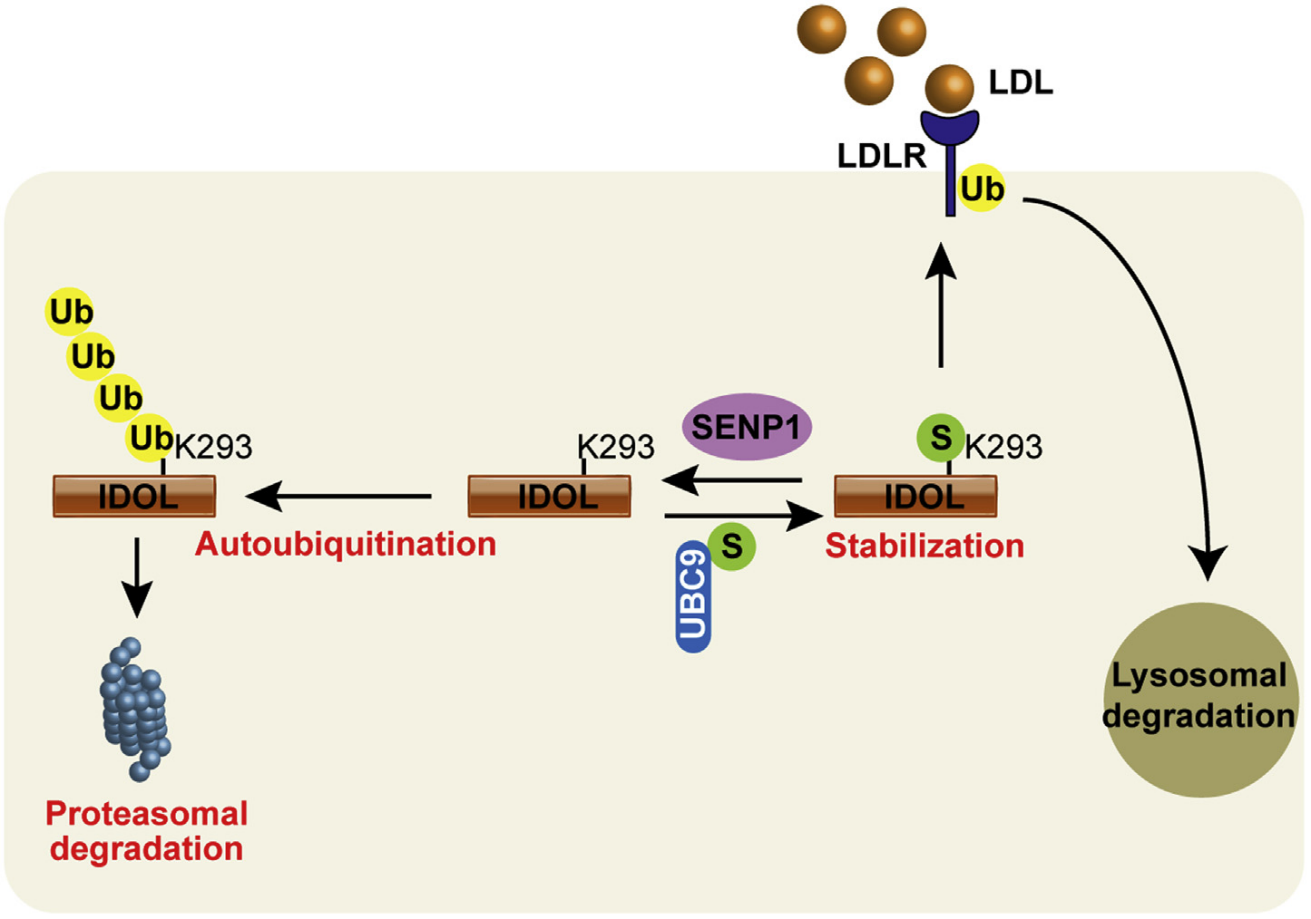
**Working model showing the function of IDOL SUMOylation in the LDLR pathway.** IDOL can undergo self-catalyzed ubiquitination in which ubiquitin (Ub) moieties are covalently attached to K293 and other residues, followed by degradation in the proteasome. However, SUMO1 (S) modification of IDOL at the K293 residue attenuates its ubiquitination, thereby increasing IDOL protein levels and promoting IDOL-mediated ubiquitination of LDLR. The ubiquitinated LDLR is eventually degraded in lysosomes. SENP1 by removing SUMO1 from modified IDOL reduces IDOL protein expression and blocks LDLR degradation by IDOL. IDOL, inducible degrader of the low-density lipoprotein receptor; LDL, low-density lipoprotein; LDLR, low-density lipoprotein receptor; SUMO, small ubiquitin-like modifier.

IDOL mediates ubiquitination and degradation of LDLR, and its mutations are tightly associated with abnormal plasma LDL-C levels in human populations (13, 30, 31). So far, self-catalyzed ubiquitination has been the only known posttranslational modification of IDOL. In this study, we present evidence for the first time that IDOL can be conjugated by SUMO1 at several lysine residues including the major autoubiquitination site K293 (Fig. 1). SUMOylation elevates IDOL protein levels by competing against its autoubiquitination (Fig. 2). SENP1 can reverse IDOL SUMOylation and reduce IDOL protein abundance (Fig. 3). This SENP1-mediated deSUMOylation of IDOL attenuates its potency in degrading LDLR and, consequently, increases LDLR expression and LDL endocytosis (Figs. 4 and 5). The proposed model for SUMOylation and SENP1-mediated deSUMOylation of IDOL in regulating the LDLR pathway is depicted in Figure 6. Our findings position SENP1 as a potential regulator of the LDLR pathway and suggest that overexpression of SENP1 may serve as a therapeutic strategy for the treatment of hypercholesterolemia.

IDOL is a well-characterized transcriptional target of the liver X receptors (LXRs) (8, 32) and, as shown here, can be modified by SUMO. Interestingly, LXRs as the oxysterol-sensitive nuclear receptors become SUMOylated in the presence of endogenous and/or synthetic ligands, thereby repressing the transcription of inflammatory target genes in multiple cell types (33, 34, 35). This raises an interesting possibility that SUMOylation of LXRs may regulate SUMOylation and expression of IDOL. However, GW3965 treatment, albeit inducing the transcription of LXR target genes including *IDOL* (Fig. S2*A*), had no effects on SUMOylation or protein levels of ectopically expressed IDOL (Fig. S2, *B*–*C*). In addition, the mRNA levels of *IDOL* remained unaltered upon overexpression, knockdown, or knockout of *SENP1* (Figs. 4*E* and 5, *B* and *F*). These results suggest that SUMOylation of IDOL and LXRs are independent of each other.

SUMOylation can regulate the activity of several E3 ubiquitin ligases. For examples, BRCA1, HERC2, and RNF168 are SUMOylated in response to genotoxic stress for increased ligase activity so as to support DNA damage repair (36, 37). SUMOylation also enhances the ability of SMURF2 to degrade transforming growth factor β, a cytokine that normally promotes mammary epithelial morphogenesis (38). By contrast, Rsp5p as the only member of the Nedd4 family of HECT-type ubiquitin ligases in yeast exhibits reduced activity upon SUMOylation, which eventually affects ubiquitin-mediated endocytosis of the manganese transporter Smf1p (39). We hereby identify one additional example of mammalian RING type ubiquitin ligase that is subjected to SUMO regulation. SUMOylation increases IDOL protein levels, whereas SENP1-mediated deSUMOylation reverses the effect. This regulation is important because addition of SENP1 effectively attenuates LDLR degradation induced by IDOL. These results suggest that IDOL abundance and its degradative potency on LDLR are positively correlated. In support of this statement, the G51S mutation of IDOL increases IDOL protein stability and promotes IDOL-stimulated LDLR degradation (13). However, Nelson *et al.* (12) found that IDOL stabilization by USP2 decreased its ability to eliminate LDLR. Whether SUMOylation enhances the ligase activity of IDOL remains to be demonstrated directly.

The smear pattern of SUMOylated IDOL suggests that SUMO1 may be conjugated as a monomer or a polymeric chain(s) at multiple lysine residues, with the evolutionarily conserved K293 residue being the principle acceptor site (Fig. 1). Coincidently, ubiquitination of IDOL takes place primarily at K293 as well (6). The K293R mutation largely, but not completely, abrogates both forms of modifications (Fig. 1) (6). In addition, we show that IDOL SUMOylation antagonizes its ubiquitination (Fig. 2*G*). Because ubiquitination and oxidation have been shown to competitively regulate the stability of acyl-CoA:cholesterol acyltransferase 2 and insulin-induced gene 2 (40, 41), it is tempting to speculate that reciprocal regulation of IDOL expression by SUMOylation and ubiquitination may similarly constitute a mechanism conferring the responsiveness of IDOL to different stimuli. Future work is needed to identify the physiological cues triggering SUMOylation and ubiquitination of IDOL, respectively.

We show that SENP1 overexpression reduces IDOL protein abundance and accelerates LDLR-mediated uptake of LDL in cultured cells (Figs. 4 and 5). At the moment, we were not able to examine the effect of SENP1 on plasma LDL-C *in vivo* because IDOL is expressed at very low levels in mouse livers (8, 42). Further, *SENP1* mRNA and protein, despite present in many human tissues including the liver, are highly enriched in the testis (43) (www.proteinatlas.org). Polymorphisms in *SENP1* have been correlated with chronic mountain sickness (44, 45) but not with lipid-related diseases as of yet. However, our findings are still of great importance considering that IDOL deficiency facilitates LDLR-dependent clearance of ApoE and β-amyloid in the brain, thereby reducing the formation and deposition of amyloid plaques and improving cognitive function in the mouse model of Alzheimer’s disease (46, 47). Ablation of IDOL also prevents diet-induced obesity through controlling neuronal very low-density lipoprotein receptor (48), another IDOL target protein (49). It will be interesting to explore whether SENP1 can regulate IDOL-related pathways beyond LDL uptake.

## Experimental procedures

### Reagents

Lovastatin (purity ≥ 98.5%, HPLC) was from Shanghai Pharm Valley. Sodium mevalonate (#4667), anti-FLAG M2 agarose beads (#A2220), phenylmethanesulfonyl fluoride (PMSF, #P7626), protease inhibitor cocktail (#P8340), and β-mercaptoethanol (#M3148) were from Sigma-Aldrich. DiI-LDL was from Yeasen (#20614ES76). GW3965 (#10054) was from Cayman. Lipofectamine RNAiMAX (#13778150) was from ThermoFisher. SUMO1-AMC (#UL-704), ubiquitin-AFC (#U-551-050), and MG132 (#I-130) were from Boston Biochem. Puromycin (#BS111) was from Biosharp. G418 (#345810), pepstatin A (#516481), and ALLN (N-acetyl-leu-leu-norleucinal, #208719) were from Calbiochem. Ni-NTA Agarose (#30230) was from Qiagen. Linear polyethylenimine (#23966-1) was from Polysciences. FuGENE HD (#E2311) and M-MLV RTase (#M1701) were from Promega. Leupeptin (#11034626001) was from Roche. DL-Dithiothreitol (DTT, #A100281) and NP-40 (A100109) were from Sangon Biotech. Lipoprotein-deficient serum (density >1.215 g/ml) and delipidated-fetal calf serum were prepared in our laboratory as described previously (50, 51). The purified PCSK9 protein was kindly provided by Dr Yan Wang (Wuhan University).

### Plasmids

The coding region of human *SENP1* was amplified from Huh7 cells by standard PCR and cloned into the p3×Flag-CMV14, pCMV- 5×Myc or pcDNA3 vectors. The coding region of human *USP2* was amplified from Huh7 cells by standard PCR and cloned into the p3×Flag-CMV14 vector. The pCMV-LDLR-5×Myc, pCMV-IDOL-3×Flag, and pEF-HA-ubiquitin were generated as described previously (13, 52). The pCMV-UBC9-5×Myc, pRK-6×His-SUMO1/2/3, and pCMV-USP19-3×Flag were kindly provided by Drs Hong-Bing Shu and Bo Zhong (Wuhan University). The mutant forms of SENP1 (C603S) and IDOL (K20R, K293R, K20R/K293R) were prepared by site-directed mutagenesis using KOD Hot Start DNA polymerase (#KOD-401; TOYOBO).

### Antibodies

The following primary antibodies were used: mouse monoclonal anti-β-actin (#A5441; Sigma), mouse monoclonal anti-FLAG (#F3165; Sigma), mouse monoclonal anti-His (#66005-1-Ig; ProteinTech), and mouse monoclonal anti-SENP1 (#sc-271360; Santa Cruz). The monoclonal antibody against c-Myc was prepared from hybridomas (ATCC, Clone 9E10). The polyclonal antibody against LDLR was generated by immunizing rabbits with the recombinant fragment corresponding to human LDLR (amino acids 98–147) followed by affinity purification with antigens. Horseradish peroxidase-conjugated goat anti-rabbit (#31460) and donkey anti-mouse (#715-035-150) IgG antibodies were from Pierce and Jackson ImmunoResearch laboratories, respectively.

### Cell culture

Huh7 (a human hepatocarcinoma cell line), CRL1601 (a McArdle RH7777 rat hepatoma cell line), and HEK293T were grown in a monolayer at 37 °C with 5% CO_2_. Cells were maintained in medium A (Dulbecco's Modified Eagle Medium supplemented with 100 units/ml of penicillin and 100 μg/ml streptomycin sulfate). Cholesterol depletion medium was medium A supplemented with 5% lipoprotein-deficient serum, 1 μM lovastatin, and 10 μM mevalonate.

### Generation of the cell lines

CRL1601 cells stably expressing SENP1 (SENP1-OE) were generated as described previously (53). In brief, CRL1601 cells were transiently transfected with pCMV-SENP1-3×Flag using FuGENE HD. After 48 h, cells were switched to medium A supplemented with 10% fetal bovine serum and 400 μg/ml G418. Medium was replaced every 2 days until single colonies were formed.

*SENP1* and *IDOL* knockout cell lines were generated using the CRISPR/Cas9 technique. The sgRNA sequences (*SENP1*, 5′-GGCAAGGACATTTGGACCGA-3′; *IDOL*, 5′-GTTGAGGCAGTCCTCGCCGT-3′) were cloned into pX330-U6-Chimeric_BB-CBh-hSpCas9 vector (#42230; Addgene). Vectors and pLKO.1 were co-transfected into Huh7 cells. Cells were grown in medium A supplemented with 10% fetal bovine serum and 2 μg/ml puromycin until single colonies were formed.

### SUMOylation assay

Huh7 cells were set up in 60-mm dishes at the density of 6 × 10^5^ on day 0 and treated as indicated in the relevant figure legends. On the day of harvesting, a quarter of cells was lysed in RIPA buffer (50 mM Tris-HCl, pH 8.0, 150 mM NaCl, 2 mM MgCl_2_, 0.1% SDS, 1.5% NP-40, 0.5% sodium deoxycholate) supplemented with protease inhibitors (1 mM PMSF, 5 μM MG132, 10 μg/ml leupeptin, 5 μg/ml pepstain A, 0.25 mM DTT, 25 μg/ml ALLN) to verify the expression of transfected plasmids, and the rest was lysed in cell lysis buffer (6 M guanidinium-HCl, 10 mM Tris-HCl, pH 8.0, 0.1 M Na_2_HPO_4_/NaH_2_PO_4_, 10 mM β-mercaptoethanol and 5 mM imidazole) to detect the SUMOylation of IDOL. After centrifugation at 14,000*g* at 4 °C for 20 min, supernatants were collected and incubated with Ni-NTA beads at 4 °C overnight. Beads were sequentially washed with cell lysis buffer supplemented with pH 8.0 wash buffer (8 M Urea, 10 mM Tris-HCl, 0.1 M Na_2_HPO_4_/NaH_2_PO_4_, 10 mM β-mercaptoethanol) and pH 6.3 wash buffer (8 M Urea, 10 mM Tris-HCl, 0.1 M Na_2_HPO_4_/NaH_2_PO_4_, 10 mM β-mercaptoethanol). Beads were then incubated with 100 μl 1× loading buffer diluted in RIPA for 10 min at 95 °C. Supernatants were collected and subjected to immunoblotting analysis.

### Ubiquitination assay

Huh7 cells were set up in 60-mm dishes at the density of 6 × 10^5^ on day 0 and treated as indicated in the figure legends. On the day of harvesting, cells were lysed in immunoprecipitation (IP) buffer (1× PBS, 1% NP-40, 1% sodium deoxycholate, 5 mM EDTA, 5 mM EGTA, 10 μg/ml leupeptin, 25 μg/ml ALLN, 5 μg/ml pepstatin A, 5 μM MG132). After centrifugation at 14,000*g* at 4 °C for 20 min, supernatants were collected and incubated with anti-FLAG M2 agarose beads at 4 °C overnight. Beads were washed with IP buffer supplemented with the abovementioned protease inhibitors (except for PMSF and DTT) for three times, and then boiled with 1× loading buffer (without β-mercaptoethanol) at 95 °C for 10 min. Supernatants were collected and subjected to immunoblotting analysis.

### Immunoblotting

Cells were harvested and lysed in 120 μl of RIPA buffer supplemented with protease inhibitors. The protein concentration of lysates was determined using the BCA kit (ThermoFisher Scientific). Lysates were mixed with SDS loading buffer (1% SDS, 6% β-mercaptoethanol, 30% glycerol and 0.0025% bromophenol blue) and boiled at 95 °C for 10 min. Proteins were resolved by SDS-PAGE and transferred to polyvinylidene fluoride membranes. Blots were blocked with 5% skim milk in TBS plus 0.075% Tween (TBST) for 1 h at room temperature and incubated with primary antibodies overnight at 4 °C. Blots were then washed with TBST for three times and incubated with secondary antibodies for 1 h at room temperature. Blots were developed with SuperSignal chemiluminescent substrate (Thermo Scientiﬁc), and densitometry was quantiﬁed using ImageJ software.

### SUMO1-AMC and Ub-AFC assays

HEK293T cells were transfected with the plasmids expressing pCMV-SENP1-3×Flag or pCMV-USP19-3×Flag. After 48 h, cells were harvested and subjected to IP using anti-FLAG M2 agarose beads. After four washes with IP buffer, beads were incubated with 3× FLAG peptide for 30 min at room temperature. The purified recombinant proteins were split into three aliquots: one for immunoblotting with the anti-FLAG antibody, one for SUMO1-AMC assay, and one for Ub-AFC assay.

For SUMO1-AMC assay, 50 nM recombinant SENP1 or USP19 protein was mixed with 1 μM SUMO1-AMC and reaction buffer A (50 mM HEPES, pH 7.5, 1 mg/ml bovine serum albumin, 1 mM MgCl_2_, 1 mM ZnCl_2_) to a total volume of 200 μl and incubated at 37 °C for 1 h. The activity of SENP1 or USP19 was determined by monitoring the released fluorescence of free AMC using an Enzyme Labeling Instrument (excitation wavelength: 380 nm; emission wavelength: 460 nm).

For Ub-AFC assay, 50 nM of SENP1 or USP19 was mixed with 0.5 μM Ub-AFC and reaction buffer B (50 mM Tris-HCl, pH 7.4, 20 mM KCl, 5 mM MgCl_2_, 1 mM DTT) to a total volume of 200 μl and incubated at 37 °C for 1 h. The activity of SENP1 or USP19 was determined by monitoring the released fluorescence of free AFC using an Enzyme Labeling Instrument (excitation wavelength: 400 nm; emission wavelength: 505 nm).

### RNA interference

Duplexes of siRNA were synthesized by Ribobio (Guangzhou, China). The siRNAs targeting human *SENP1* (*SENP1*-1, 5′- CCAAGCUAUUACUCAGAUA-3′; *SENP1*-2, 5′-AACUACAUCUUCGUGUACCUC-3′) were transfected into Huh7 cells using Lipofectamine RNAiMAX according to the manufacturer’ instructions.

### RNA extraction and real-time PCR

Total RNA was extracted from cells using TRIzol reagent (#T9424; Sigma) and reversely transcribed using oligo dTs and M-MLV RTase (Promega). The cDNAs were subjected to quantitative real-time PCR using a BioRad CFX96 Real-Time System as previously described (54). The human primers were as follows: *SENP1* (forward, 5′-CGGTTCCGGTTCGGACTTTG-3′, reverse, 5′-CGAAAGCTGGTCCTCTGGAA-3′); *GAPDH* (forward, 5′-AGAAGGCTGGGGCTCATTTG-3′, reverse, 5′-AGGGGCCATCCACAGTCTTC-3′); *IDOL* (forward, 5′-CCCCAGCCATGCTGTGTTAT-3′, reverse, 5′-GCCTGCACACCTGGTTGA-3′); *LDLR* (forward, 5′-CTTGGAGGATGAAAAGAGGCTGG-3′, reverse, 5′-CTGGGTGAGGTTGTGGAAGAGAA-3′); *LXRα* (forward, 5′-TGGACACCTACATGCGTCGCAA-3′, reverse, 5′-CAAGGATGTGGCATGAGCCTGT-3′); *ABCA1* (forward, 5′-CAGGCTACTACCTGACCTTGGT-3′, reverse, 5′-CTGCTCTGAGAAACACTGTCCTC-3′); *SREBP-1C* (forward, 5′-GGATTGCACTTTCGAAGACATG-3′, reverse, 5′-AGGATGCTCAGTGGCACTG-3′). The rat primers were as follows: *Senp1* (forward, 5′-CGCCAGATTGAAGAGCAGA-3′, reverse, 5′-AGAGGAACACGAAGGTGGAG-3′); *Ldlr* (forward, 5′-GATTGGCTATGAGTGCCTATGTC-3′, reverse, 5′-GTGAAGAGCAGAAACCCTATGG-3′); *Idol* (forward, 5′-TACAGGAGCAGACAAGGCAT-3′, reverse, 5′-AGGGCACTAAGTTCCACTGC-3′); *Gapdh* (forward, 5′-TCACCATCTTCCAGGAGCGA-3′, reverse, 5′-GATGGGGACTCCTCAGCAAC-3′).

### DiI-LDL endocytosis assay

SENP1-OE cells, *SENP1* knockout cells, and the corresponding control cells were seeded on glass coverslips and incubated in cholesterol depletion medium at 4 °C for 30 min. Cells were then exposed to 10 μg/ml DiI-LDL diluted in cholesterol depletion medium at 4 °C for 1 h. After two PBS washes, cells were switched to 37 °C and grown in fresh cholesterol depletion medium for various durations. Cells were finally fixed with 4% paraformaldehyde, washed with PBS and imaged under a Leica SP8 confocal microscope. The relative mean fluorescence intensity of internalized DiI-LDL was quantified by Image J as previously described (55).

### Statistical analysis

Statistical analyses were performed using GraphPad Prism 7 software. Data are presented as mean ± SEM and analyzed by unpaired two-tailed Student’s *t* test or one-way ANOVA as indicated in the relevant figure legends. Statistical significance was set at *p* < 0.05.

## Data availability

All relevant data are within the manuscript and its Supporting Information files.

## Conflict of interest

The authors declare that they have no conflicts of interest with the contents of this article.
