# Supplementary material for: SUMOylation of the ubiquitin ligase IDOL decreases LDL receptor levels and is reversed by SENP1
Source: J Biol Chem. 2020 Nov 23;296:100032. doi: 10.1074/jbc.RA120.015420 (PMC7948399; doi:10.1074/jbc.RA120.015420)
Supplement: Figures S1 and S2 [file mmc1.pdf]

SUMOylation of the ubiquitin ligase IDOL decreases LDL receptor levels and is reversed by SENP1

Ju-Qiong Wang[1], Zi-Cun Lin[1], Liang-Liang Li[1], Shao-Fang Zhang[1], Wei-Hui Li[1], Wei Liu[1],

Bao-Liang Song[1], Jie Luo[1,†]

List of Supporting Information

Figure S1. PCSK9-induced LDLR degradation is not altered by SENP1 overexpression.
Figure S2. LXR agonist effectively induces mRNA expression but neither SUMOylation nor protein expression of IDOL.

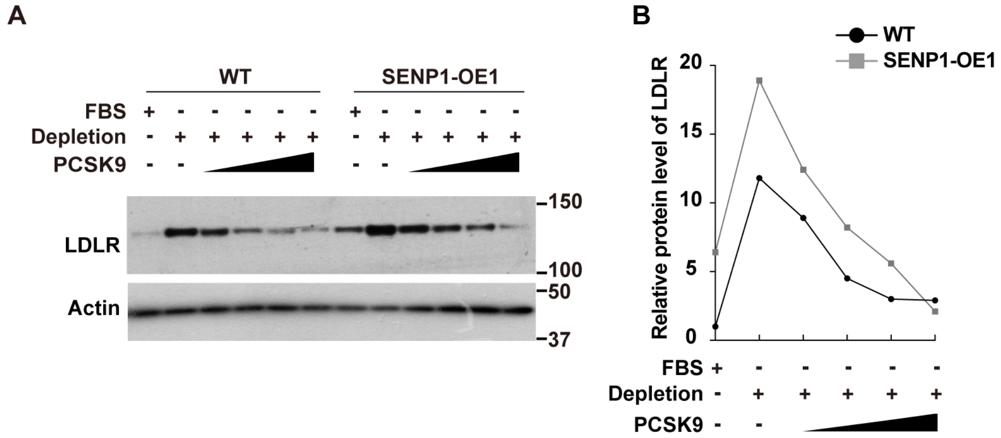

Figure S1. PCSK9-induced LDLR degradation is not altered by SENP1 overexpression. CRL1601 and SENP1-OE1 cells were incubated with fetal bovine serum (FBS)-containing medium or cholesterol depletion medium (medium A plus 5% lipoprotein-deficient serum, 1 μM lovastatin and 10 μM mevalonate) in the absence or presence of increasing concentrations of PCSK9 for 4 h. After 48 h, cells were harvested for immunoblotting analysis (*A*). Densitometric analysis of the endogenous LDLR protein is shown in (*B*). The densitometry of LDLR protein in CRL1601 cells grown in FBS condition is defined as 1. Results are from a single experiment.

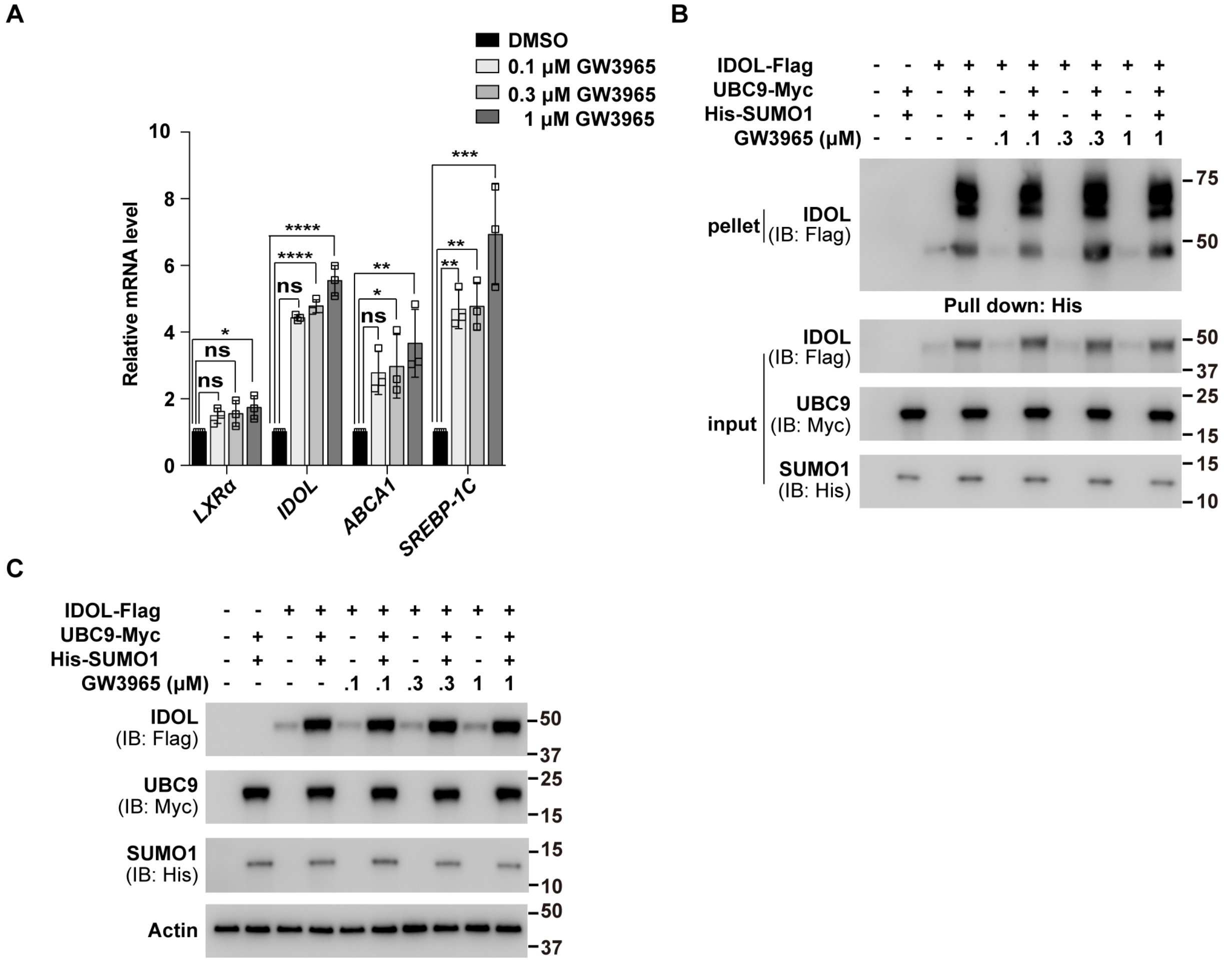

Figure S2. LXR agonist effectively induces mRNA expression but neither SUMOylation nor protein expression of IDOL. *A,* Huh7 cells were incubated in the medium containing 5% delipidated-fetal calf serum, 1 μM lovastatin and 10 μM mevalonate and the indicated concentrations of GW3965 for 16 h. Cells were then harvested for RT-qPCR analysis. Data are normalized to DMSO-treated cells and presented as mean ± SEM (n = 3 independent experiments). $^{*}p< 0.05$, $^{**}p< 0.01$, $^{***}p< 0.001$, $^{****}p< 0.0001$, ns, no significance. One-way ANOVA. *B-C*, Huh7 cells were transfected as indicated and treated as described in (*A*) for 16 h. Cells were then harvested for the SUMOylation assay (*B*) and immunoblotting (*C*). Results in (*B*) and (*C*) are representative of two independent experiments.
